# Supplementary material for: Sales forecasting for retail stores using hybrid neural networks and sales-affecting variables
Source: PeerJ Comput Sci. 2025 Sep 11;11:e3058. doi: 10.7717/peerj-cs.3058 (PMC12453866; doi:10.7717/peerj-cs.3058)
Supplement: Supplemental Information 1 [file peerj-cs-11-3058-s001.rtf]

Sales Forecasting for Retail Stores using Hybrid Neural Network and Sales affecting VariablesOverviewThis project implements a hybrid Neural Network model combining Long Short-Term Memory (LSTM) and Convolutional Neural Networks (CNN) for sales forecasting. The model is trained on historical sales data, incorporating various external factors such as weather conditions, events, and lagged sales to improve prediction accuracy.Features	•Data Preprocessing & Feature Engineering: Extracts time-based features, normalizes data, and applies one-hot encoding for categorical variables.	•LSTM & CNN Hybrid Model: Uses CNN for feature extraction and LSTM for sequence learning.	•Performance Metrics: Calculates MAE, RMSE, and MAPE to evaluate model performance.	•Visualization: Plots training history and forecast results.	•CSV Export: Saves the forecasted results into forecast_sales_results.csv.Dataset	•The dataset is loaded from Sales_Data_Fsd_All.csv.	•It includes sales data along with external influencing factors.	•Ensure that the dataset is placed in the correct directory before running the script. Modifying the following line in source code:	⁃df = pd.read_csv("/content/sample_data/Sales_Data_Fsd_All.csv")Installation & RequirementsPrerequisitesIf you are running source code on your local machine, make sure you have the following dependencies installed:	•Python 3.x	•TensorFlow	•NumPy	•Pandas	•Matplotlib	•Scikit-learnInstallationpip install tensorflow numpy pandas matplotlib scikit-learnOtherwise, the code can be directly runnable using a Google Colab notebook. No need to install above librarries in thais case.Usage1. Load the datasetEnsure the dataset is correctly formatted and contains required fields (Sale, Date, Status, etc.).2. Run the scriptExecute the Python script to train the model and generate forecasts.python sales_forecast.py3. View Results	•Forecasted sales are saved in forecast_sales_results.csv.	•Graphical results are displayed showing the original vs. predicted sales.Model Architecture	•CNN Layer: Extracts spatial features from the input sequence.	•MaxPooling Layer: Reduces dimensionality while preserving important features.	•LSTM Layer: Learns sequential dependencies in the sales data.	•Dense Layer: Produces the final sales forecast.Performance EvaluationThe model evaluates performance using the following metrics:	•Mean Absolute Error (MAE)	•Root Mean Squared Error (RMSE)	•Mean Absolute Percentage Error (MAPE)Results	•The model successfully predicts sales trends with reasonable accuracy.	•Predictions are visualized and compared to actual sales data.ContributionsFeel free to make chahnges in the code to improve the model.AcknowledgmentsSpecial thanks to the data providers and TensorFlow community for their contributions.
