# Supplementary material for: Sales forecasting for retail stores using hybrid neural networks and sales-affecting variables
Source: PeerJ Comput Sci. 2025 Sep 11;11:e3058. doi: 10.7717/peerj-cs.3058 (PMC12453866; doi:10.7717/peerj-cs.3058)
Supplement: Supplemental Information 2 [file peerj-cs-11-3058-s002.py]

#### Final working code LSTM + CNNimport numpy as npfrom tensorflow.keras.models import Sequentialfrom tensorflow.keras.layers import Conv1D, MaxPooling1D, LSTM, Densefrom tensorflow.keras.layers import Dense, Dropoutimport pandas as pdfrom matplotlib import pyplot as pltfrom sklearn.preprocessing import StandardScaler#from datetime import datetime# Load the datasetdf = pd.read_csv("/content/sample_data/Sales_Data_Fsd_All.csv")# Convert 'Date' column to datetimedf['Date'] = pd.to_datetime(df['Date'], format='mixed')df['DayOfWeek'] = df['Date'].dt.dayofweekdf['Month'] = df['Date'].dt.monthdf['DaySin'] = np.sin(2 * np.pi * df['DayOfWeek'] / 7)df['DayCos'] = np.cos(2 * np.pi * df['DayOfWeek'] / 7)# Feature Engineering (Example: Lagged Sales)df['Lagged_Sales'] = df['Sale'].shift(1)df = df.dropna()  # Remove rows with NaN values after shiftingdf['Sales_Lag7'] = df['Sale'].shift(7)df['Sales_MA_7'] = df['Sale'].rolling(window=7).mean()df = df.dropna()  # Remove NaN values after calculating moving averagedf['Sales_Std7'] = df['Sale'].rolling(window=7).std()# Calculate lagged differencesdf['Sales_Diff'] = df['Sale'] - df['Sale'].shift(1)df = df.dropna()# Create day of the week feature (0 = Monday, 6 = Sunday)df['Day_of_Week11'] = df['Date'].dt.dayofweek  # Create 'Day_of_Week11' before selecting features# Select features for the modelfeatures = ['Sale', 'Lagged_Sales', 'DaySin', 'DayCos', 'Sales_Lag7', 'Status', 'EffectiveRain', 'Sales_MA_7', 'EffectiveTemperature', 'Sales_Diff', 'Sales_Std7', 'Day_of_Week11', 'PositiveEvents', 'NegativeEvents']data = df[features]# One-hot encode day of the week to add categorical weekday effectdata = pd.get_dummies(data, columns=['Day_of_Week11'], drop_first=True) # One-hot encode after selecting featuresdata = data.values # Convert to NumPy array after value replacement#Variables for trainingcols = list(df)[1:13]print(cols)#New dataframe with only training data - 5 columnsdf_for_training = df[cols]# Convert 'Status' values in df_for_training to numeric representations:df_for_training['Status'].replace(['OPEN', 'CLOSED'], [0, 1], inplace=True)# Convert 'EffectiveRain values within the DataFramedf_for_training['EffectiveRain'].replace(['No', 'EffectiveRain'],                        [0, 1], inplace=True)# Convert 'EffectiveTemperature values within the DataFramedf_for_training['EffectiveTemperature'].replace(['No', 'HotWeather'],                        [0, 1], inplace=True)# Convert 'PositiveEvents values within the DataFramedf_for_training['PositiveEvents'].replace(['No', 'SalaryDay', 'Holiday', 'Promotions', 'Event'],                        [0, 1, 2, 3, 4], inplace=True)# Convert 'NegativeEvents values within the DataFramedf_for_training['NegativeEvents'].replace(['No', 'Wednesday', 'Protest', 'PartiallyClosed'],                        [0, 1, 2, 3], inplace=True)df_for_training = df_for_training.astype(int)#LSTM uses sigmoid and tanh that are sensitive to magnitude so values need to be normalized# normalize the datasetscaler = StandardScaler()scaler = scaler.fit(df_for_training)df_for_training_scaled = scaler.transform(df_for_training)#Empty lists to be populated using formatted training datatrainX = []trainY = []n_future = 1   # Number of days we want to look into the future based on the past days.n_past = 60  # Number of past days we want to use to predict the future.for i in range(n_past, len(df_for_training_scaled) - n_future +1):    trainX.append(df_for_training_scaled[i - n_past:i, 0:df_for_training.shape[1]])    trainY.append(df_for_training_scaled[i + n_future - 1:i + n_future, 0])trainX, trainY = np.array(trainX), np.array(trainY)print('trainX shape == {}.'.format(trainX.shape))print('trainY shape == {}.'.format(trainY.shape))# define the Autoencoder modelmodel = Sequential()# LSTM + CNNmodel.add(Conv1D(filters=64, kernel_size=4, activation='relu', input_shape=(trainX.shape[1], trainX.shape[2])))  # CNN layermodel.add(MaxPooling1D(pool_size=2))  # Pooling layermodel.add(LSTM(50, return_sequences=False))  # LSTM layermodel.add(Dense(trainY.shape[1]))model.compile(optimizer='adam', loss='mse')model.summary()# fit the modelhistory = model.fit(trainX, trainY, epochs=150, batch_size=32, validation_split=0.1, verbose=1)plt.plot(history.history['loss'], label='Training loss')plt.plot(history.history['val_loss'], label='Validation loss')plt.legend()n_past = 60n_days_for_prediction=60  #let us predict past 60 days# Get training dates from the 'Date' column of the original DataFrametrain_dates = df['Date']  # Assuming 'Date' column contains datetime objectspredict_period_dates = pd.date_range(list(train_dates)[-n_past], periods=n_days_for_prediction).tolist()# print(predict_period_dates)#Make predictionprediction = model.predict(trainX[-n_days_for_prediction:]) #shape = (n, 1) where n is the n_days_for_prediction#Perform inverse transformation to rescale back to original range#Since we used 5 variables for transform, the inverse expects same dimensions#Therefore, let us copy our values 5 times and discard them after inverse transformprediction_copies = np.repeat(prediction, df_for_training.shape[1], axis=-1)y_pred_future = scaler.inverse_transform(prediction_copies)[:,0]# Convert timestamp to dateforecast_dates = []for time_i in predict_period_dates:    forecast_dates.append(time_i.date())# Get the last 'n_days_for_prediction' values from the 'Sale' columnoriginal_sales = df['Sale'].tail(n_days_for_prediction).valuesdf_forecast = pd.DataFrame({'Date': np.array(forecast_dates),                            'Sale': y_pred_future.astype(int),                            'Original': original_sales})df_forecast['Date']=pd.to_datetime(df_forecast['Date'])# Calculate Mean Absolute Error (MAE)mae = np.mean(np.abs(df_forecast['Sale'] - df_forecast['Original']))# Calculate Root Mean Squared Error (RMSE)rmse = np.sqrt(np.mean((df_forecast['Sale'] - df_forecast['Original'])**2))# Calculate Mean Absolute Percentage Error (MAPE)mape = np.mean(np.abs((df_forecast['Sale'] - df_forecast['Original']) / df_forecast['Original'])) * 100# Print the resultsprint(f"MAE: {mae}")print(f"RMSE: {rmse}")print(f"MAPE: {mape:.2f}%")original = df[['Date', 'Sale']]original['Date']=pd.to_datetime(original['Date'])# original = original.loc[original['Date'] >= '2020-5-1']print(df_forecast)df_forecast.to_csv('forecast_sales_results.csv', index=False)# Plotting the forecastplt.figure(figsize=(12, 6))  # Adjust figure size if neededplt.plot(df_forecast['Date'], df_forecast['Sale'], label='Forecast')plt.plot(df_forecast['Date'], df_forecast['Original'], label='Original')plt.xlabel('Date')plt.ylabel('Sale')plt.title('Sales Forecast vs. Original Using LSTM and CNN together')plt.legend()plt.grid(True)plt.show()
