# Supplementary material for: Sales forecasting for retail stores using hybrid neural networks and sales-affecting variables
Source: PeerJ Comput Sci. 2025 Sep 11;11:e3058. doi: 10.7717/peerj-cs.3058 (PMC12453866; doi:10.7717/peerj-cs.3058)
Supplement: Supplemental Information 4 [file peerj-cs-11-3058-s004.pdf]

Dear Peerj Staff,

I hope you are doing well.

We would like to inform you that Ahmed Zohier Elhendi analyzed and interpretation of the data and models, discussed the results, reviewed

drafts of the article and revised critically and approved the final draft. His contributions are:

1. After critical review he added Research Contribution section

2. Added new subsection Tools and Technologies for explaining the experimental setup.

3. He reviewed two more papers to add into the manuscript for the motivational support of hybrid model.

4. He added Figure 3 along with description for the visual evidence for the impact of demographic variables.

Please feel free to contact me if you have any questions.

Thanks and best,

Saad Mansur, Kashif Sattar, Seyed Ebrahim Hosseini, Shahbaz Pervez Chattha, Iftikhar Ahmad, Kashif Saleem, and Ahmed Zohier Elhendi
